# Supplementary figures and images for: TRPV6, TRPM6 and TRPM7 Do Not Contribute to Hair-Cell Mechanotransduction
Source: Front Cell Neurosci. 2018 Feb 20;12:41. doi: 10.3389/fncel.2018.00041 (PMC5826258; doi:10.3389/fncel.2018.00041)

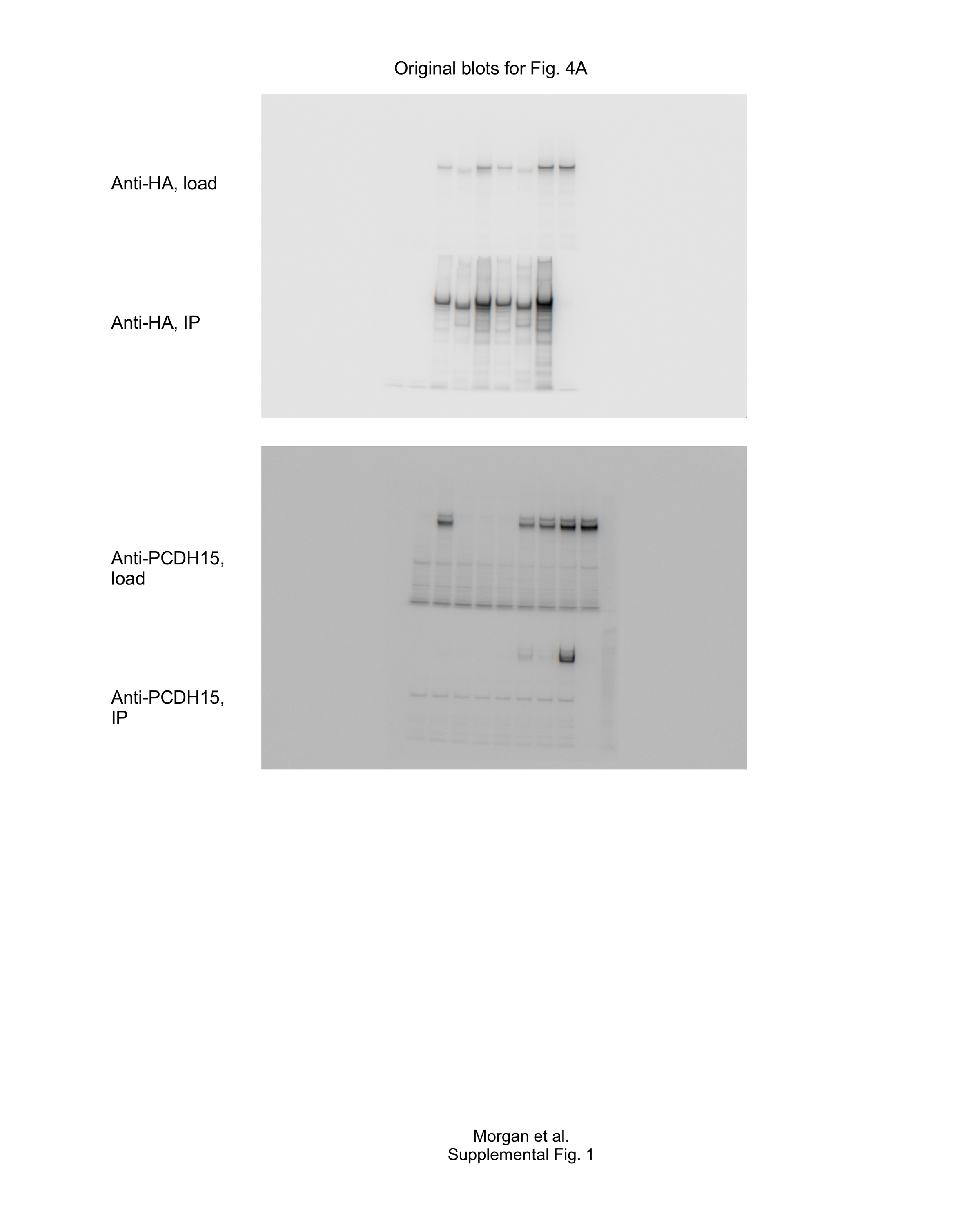

Supplement: Supplementary file 1 [file Image_1.tif]

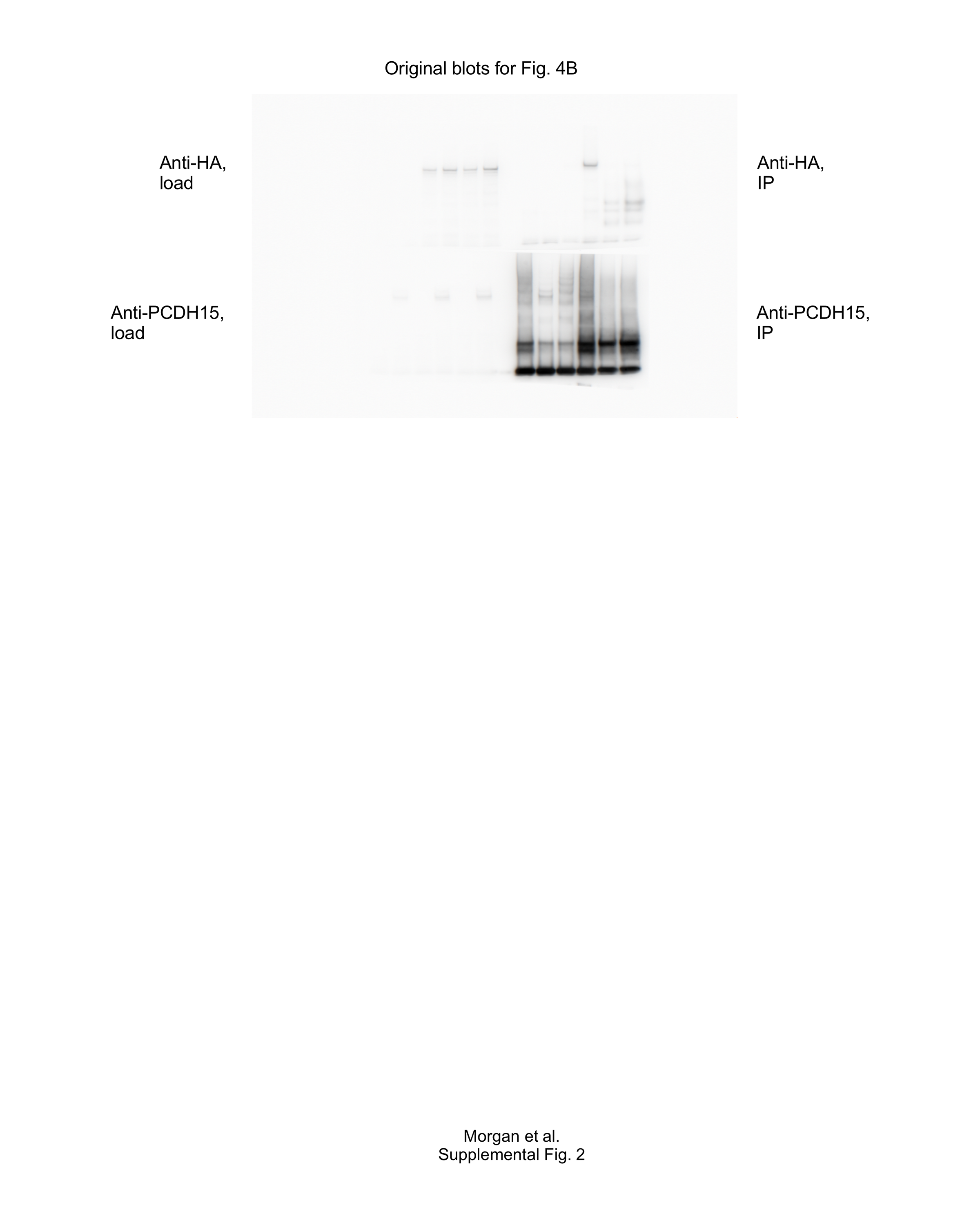

Supplement: Supplementary file 2 [file Image_2.tif]

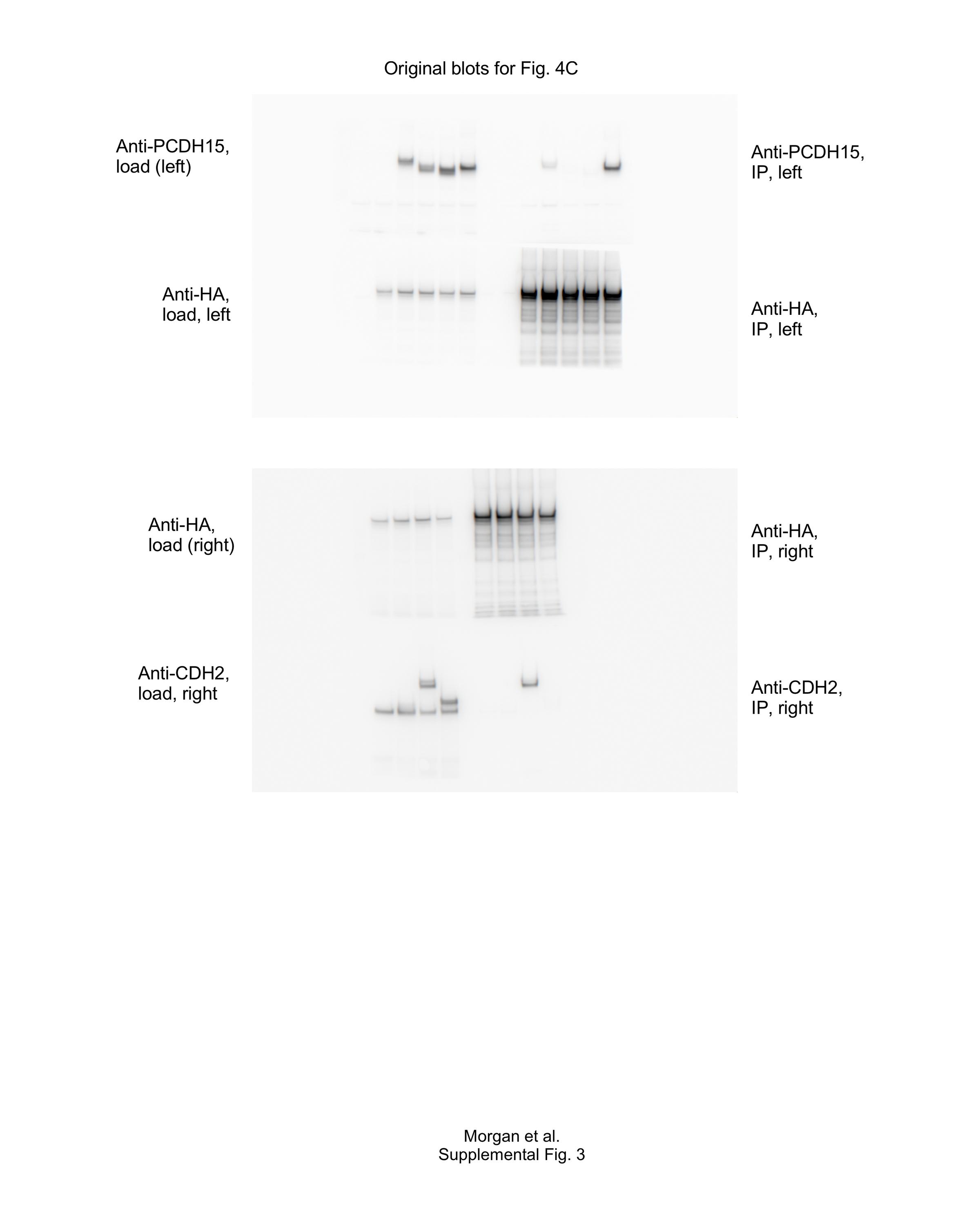

Supplement: Supplementary file 3 [file Image_3.tif]

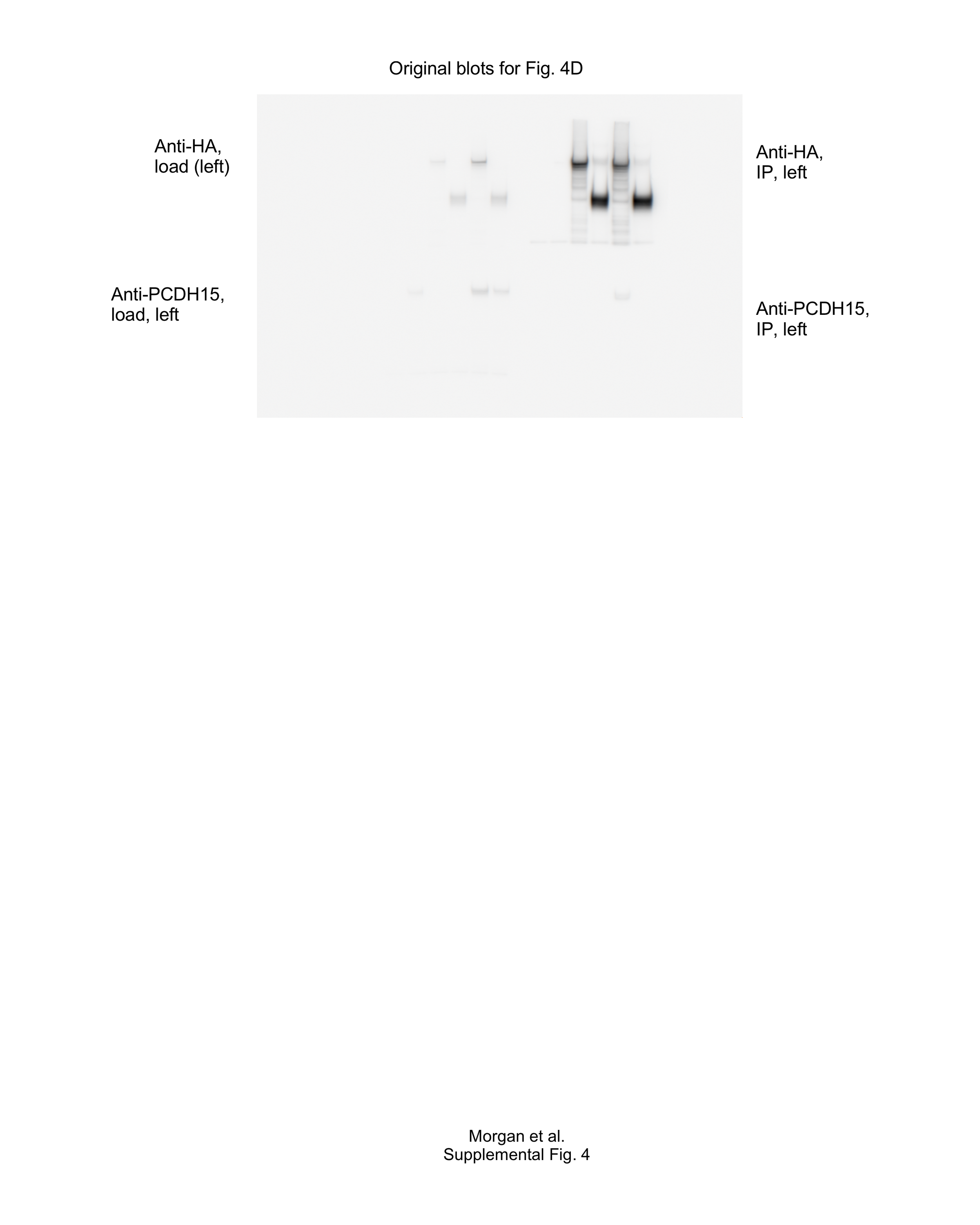

Supplement: Supplementary file 4 [file Image_4.tif]

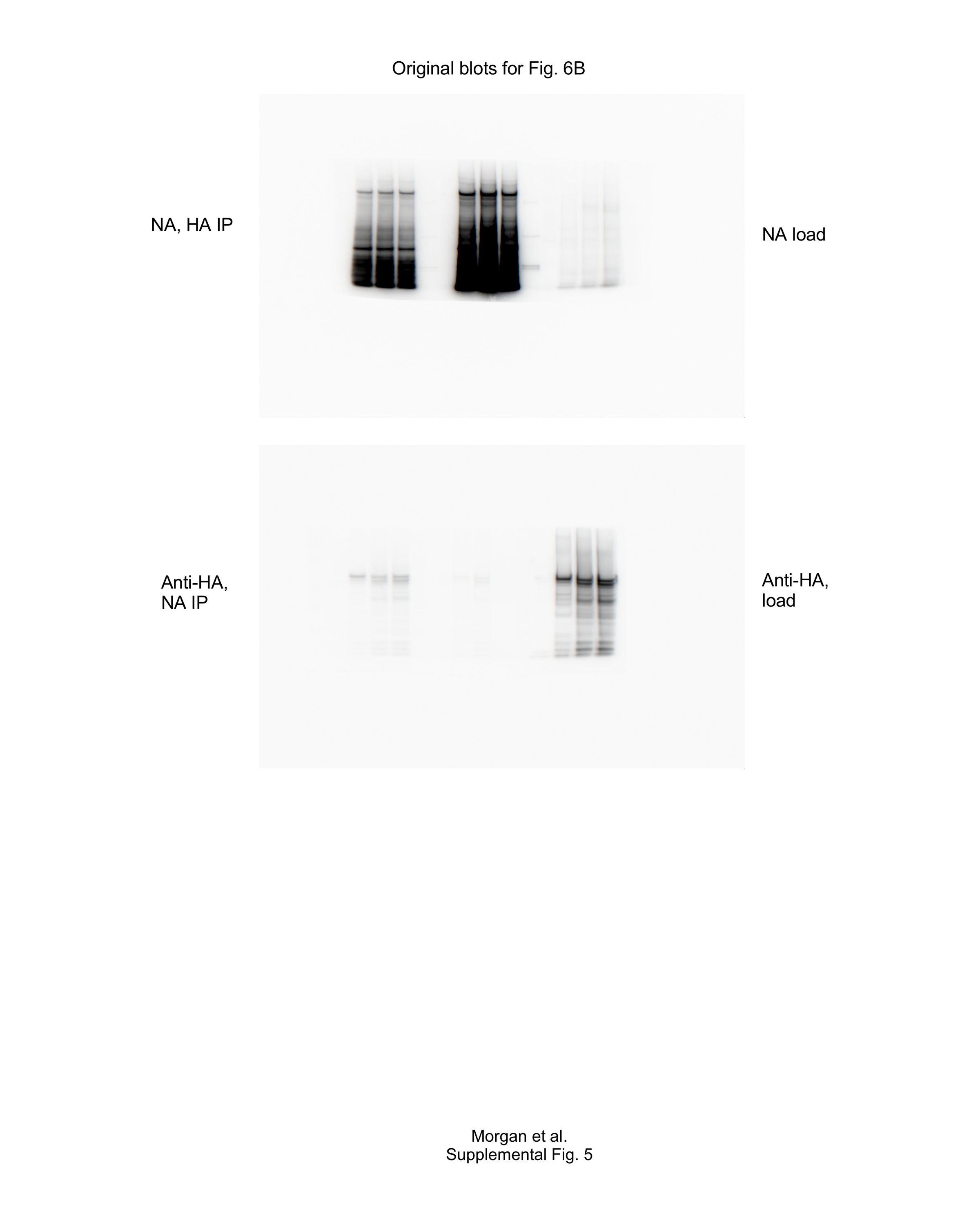

Supplement: Supplementary file 5 [file Image_5.tif]

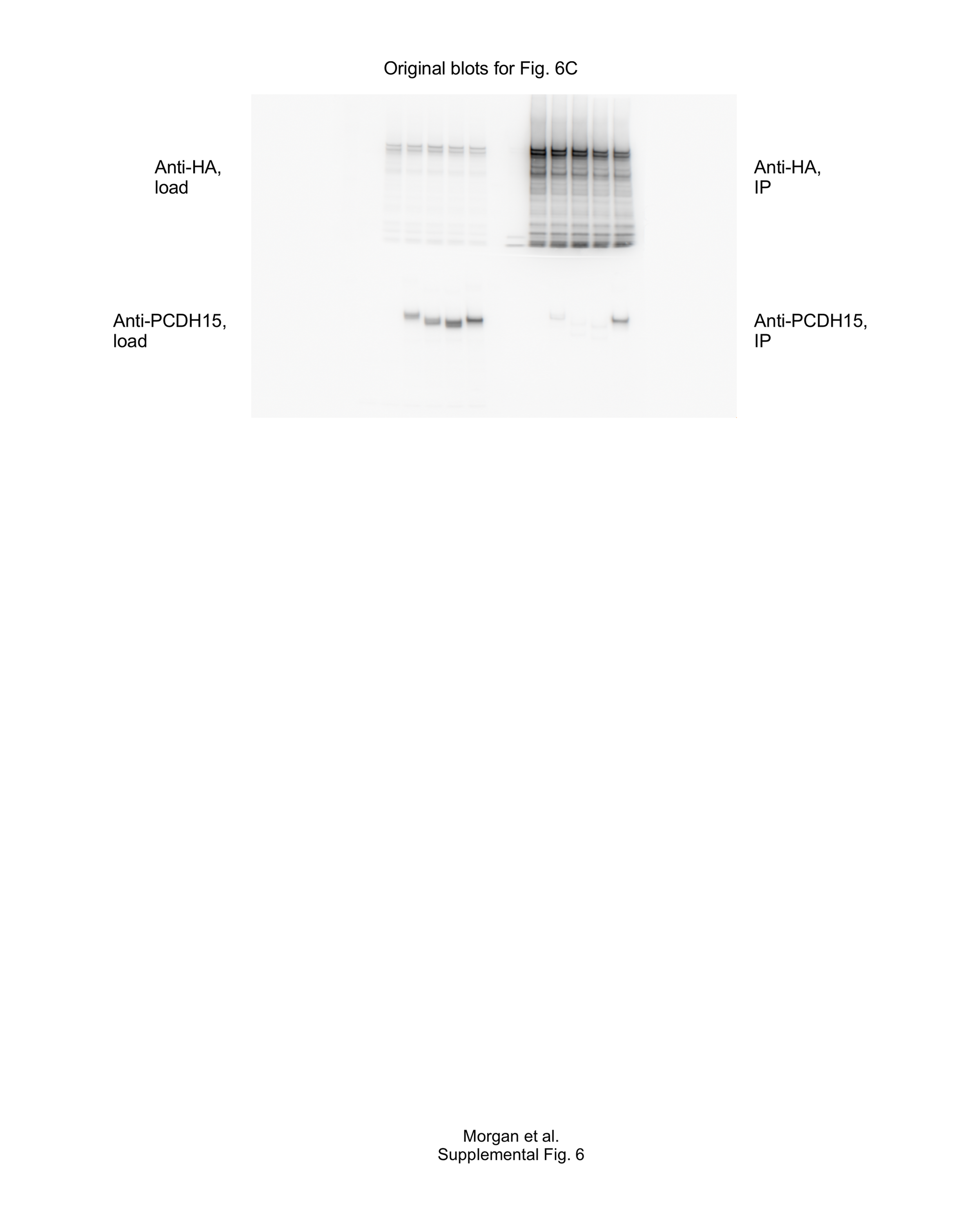

Supplement: Supplementary file 6 [file Image_6.tif]
